# 3D Spheroids Derived from Human Lipedema ASCs Demonstrated Similar Adipogenic Differentiation Potential and ECM Remodeling to Non-Lipedema ASCs In Vitro

**DOI:** 10.3390/ijms21218350

**Published:** 2020-11-07

**Authors:** Sara Al-Ghadban, India A. Pursell, Zaidmara T. Diaz, Karen L. Herbst, Bruce A. Bunnell

**Affiliations:** 1 Department of Microbiology, Immunology and Genetics, University of North Texas Health Science Center, Fort Worth, TX 76107, USA; 2 Center for Stem Cell Research and Regenerative Medicine, Tulane University School of Medicine, New Orleans, LA 70112, USA; ipursell@tulane.edu (I.A.P.); zdiaz@tulane.edu (Z.T.D.); 3 Medical Director, Limitless Therapeutics, Los Angeles, CA 90025, USA; kaherbst@gmail.com

**Keywords:** adipose tissue-derived stem cells (ASCs), lipedema, spheroids, adipogenic differentiation, ECM markers, inflammation

## Abstract

The growth and differentiation of adipose tissue-derived stem cells (ASCs) is stimulated and regulated by the adipose tissue (AT) microenvironment. In lipedema, both inflammation and hypoxia influence the expansion and differentiation of ASCs, resulting in hypertrophic adipocytes and deposition of collagen, a primary component of the extracellular matrix (ECM). The goal of this study was to characterize the adipogenic differentiation potential and assess the levels of expression of ECM-remodeling markers in 3D spheroids derived from ASCs isolated from both lipedema and healthy individuals. The data showed an increase in the expression of the adipogenic genes (ADIPOQ, LPL, PPAR-γ and Glut4), a decrease in matrix metalloproteinases (MMP2, 9 and 11), with no significant changes in the expression of ECM markers (collagen and fibronectin), or integrin A5 in 3D differentiated lipedema spheroids as compared to healthy spheroids. In addition, no statistically significant changes in the levels of expression of inflammatory genes were detected in any of the samples. However, immunofluorescence staining showed a decrease in fibronectin and increase in laminin and Collagen VI expression in the 3D differentiated spheroids in both groups. The use of 3D ASC spheroids provide a functional model to study the cellular and molecular characteristics of lipedema AT.

## 1. Introduction

Adipose tissue-derived stem cells (ASCs) are immunologically privileged cells that have been extensively studied in regenerative medicine and tissue engineering. ASCs have been implicated in the treatment of many pathological and chronic disorders such as diabetes, multiple sclerosis, neurodegenerative, Crohn’s and Graft-versus-host disease; however, the source of the stem cells should be carefully considered for successful outcomes. Studies have shown that ASCs isolated from individuals with metabolic disorders such as obesity or type 2 diabetes demonstrated a lower immunoregulatory potential, reduced self-renewal and differentiation capacity compared to ASCs derived from healthy lean donors. Thus, a full characterization of ASCs derived from lipedema patients is significant to establish a model for the disease for pre-clinical studies [1,2,3,4,5,6,7,8,9]. 

ASCs, isolated from the stromal vascular fraction (SVF) of adipose tissue (AT), are typically cultured in two-dimensional (2D) monolayers using standard techniques [10,11]. Researchers have recently shifted their focus from studying ASCs in 2D to three-dimensional (3D) cultures as the latter showed higher therapeutic potential and enhanced differentiation capabilities [12,13,14,15,16,17]. Studying ASC spheroids enabled researchers to investigate ASCs in an in vivo-like cellular environment by promoting their interactions with other cells and extracellular matrix (ECM) components [18]. These interactions are essential to instigate ASC differentiation into specific cell lineages such as adipocytes, osteocytes, and chondrocytes. In recent years, numerous techniques have been developed to generate 3D spheroids, like hanging drops and liquid overlay cultures, hydrogels, bioreactors and scaffolds [19,20,21]. All these techniques have proven successful in forming ASC spheroids alone or in co-culture models [22,23,24,25,26,27]. 

Lipedema is a connective tissue disorder that affects ~11% of women worldwide [28,29,30]. The disease is characterized by hypertrophic adipocytes, inflammation, leaky blood and lymphatic vessels and fibrosis, all of which contributed to increased proliferation and adipogenic differentiation of ASCs/preadipocytes as well as tissue remodeling [31,32,33,34]. We and others have shown that SVF, a heterogeneous population of cells, isolated from lipedema AT and expanded in monolayer cultures, is composed of significantly higher levels of the mesenchymal stem marker (CD90; cluster of differentiation 90) and endothelial markers (CD31 and CD146) as compared to individuals unaffected with lipedema [35,36], which may be a contributing factor to the leaky blood and lymphatic vessels detected in lipedema tissues. We and others have also shown an increase in fibrosis and angiogenesis in lipedema adipose tissue [32,33]. The increase in the cell number of SVF endothelial cells, elevated levels of secreted VEGF in the blood of women with lipedema [37] and the increased fibrosis and angiogenesis in lipedema thigh adipose tissue suggest that neo-vascularization in AT and the synthesis of new ECM components occurs [38,39]. Fibrous collagen (I-VI), and adhesive proteins, laminin (LN) and fibronectin (FN), are the main ECM proteins found in adipose tissue [40]. During tissue expansion, the ECM is remodeled to accommodate adipocyte hypertrophy or hyperplasia as well as increased adipogenesis; these modifications are regulated by the matrix enzymes including metalloproteinase (MMP)-2 and MMP-9 [41,42,43]. ECM receptors such as integrins, CD36 and CD44 also play a vital role in tissue remodeling and adipocyte differentiation primarily by regulating insulin sensitivity and the expression of downstream targets such as collagen VI [44]. 

In our previous work, ASCs isolated from the AT from lipedema patients and healthy individuals were characterized and demonstrated that lipedema ASCs cultured in a 2D monolayer have a higher adipogenic differentiation potential compared to healthy ASCs [36]. In this study, the goal was to characterize the adipogenic differentiation potential of the same ASC lines in 3D cultures (ASC spheroids) to gain insights into the ECM produced by ASCs induced to undergo differentiation into adipocytes. We analyzed the expression of the primary adipogenic (ADIPOQ, LPL, PPAR-γ and Glut4) and ECM-remodeling (collagen, FN, LN, ITAGA5, MMP2, 9 and 11) markers at the transcriptional level and by immunohistochemistry in 3D spheroids derived from ASCs from both lipedema and healthy individuals. The data in this study demonstrated an increase in the expression of the adipogenic genes, a decrease in matrix metalloproteinase (MMP) expression with no significant changes in the expression of ECM or inflammatory genes in 3D differentiated lipedema spheroids as compared to healthy spheroids. Additionally, a decrease in fibronectin and an increase in laminin and Col VI proteins were detected in 3D differentiated spheroids in both groups. 

## 2. Results

### 2.1. ASC Spheroid Formation and Stemness Characterization

**Figure 1:**
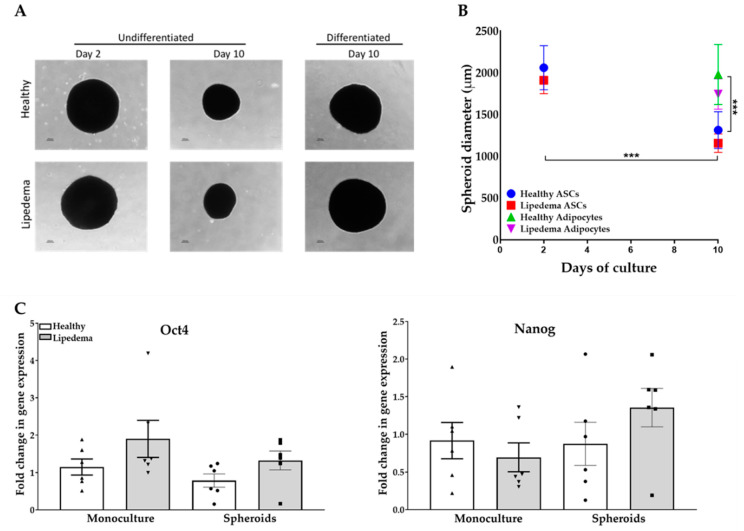
The morphology and the expression of stemness markers of adipose tissue-derived stem cell (ASC) spheroids derived from healthy and lipedema ASCs. (**A**) Representative images of self-assembled ASC spheroids observed by phase contrast microscopy at days 2 and 10 of culture (scale bar, 100 μm). (**B**) Sizes of spheroids measured using ImageJ software showing a significant reduction in diameter between undifferentiated and day ten differentiated spheroids in both groups. Data are shown as mean ± SEM (*n* = 7 per group). *** *p* < 0.001. (**C**) qRT-PCR showing the expression of stemness markers Nanog and Oct 4 in 2D monolayer culture and ASC spheroids at day 10 (*n* = 6 per group).

ASCs seeded on agarose-coated plates formed spheroids within 24 h of culture. Images of self-assembled spheroids, shown in Figure 1A, were taken at days two and ten for undifferentiated and adipogenic-induced differentiated spheroids. ASC spheroids displayed a rounded, dense aggregate structure with size ranging from 1000 to 2000 µm in diameter. At day 10, undifferentiated spheroids showed a significant decrease in diameter (*p* < 0.001) compared to day two undifferentiated and day ten differentiated spheroids in both groups (Figure 1B). Furthermore, ASC spheroids maintain their stemness while in suspension, confirmed by the expression of Nanog and Oct4 genes similarly to monolayer culture, that is imperative to their potential application in regenerative medicine (Figure 1C). It is worth noting there was no statistical difference between the monolayer 2D culture and ASC spheroids in both groups.

### 2.2. Adipogenic Differentiation of ASC-Derived Spheroids

**Figure 2:**
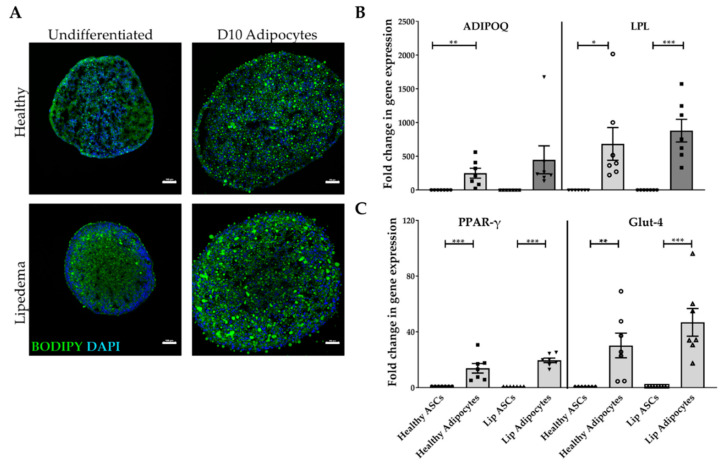
In vitro adipogenic differentiation of ASC spheroids. (**A**) BODIPY staining (green) showing lipid accumulation in differentiated adipocytes (scale bar, 100 μm). Nuclei were stained with DAPI (blue). (**B**,**C**) qRT-PCR showing the expression of ADIPOQ, LPL (**B**), PPAR-γ, Glut4 (**C**) in ASCs and adipocytes (*n* = 7 per group). qRT-PCR analysis showed a significant increase in gene expression in differentiated adipocytes. The values are the mean ± SEM. * *p* < 0.05; ** *p* < 0.01; *** *p* < 0.001. Abbreviations: ADIPOQ: adiponectin; LPL: lipoprotein lipase; PPAR-γ: peroxisome proliferator-activated receptor gamma; and Glut4: glucose transporter type 4; Lip: lipedema.

Adipogenesis of ASC spheroids was demonstrated by lipid accumulation in differentiated cells detected by BODIPY staining at day ten as compared to undifferentiated spheroids cultured in growth media of both groups (Figure 2A). BODIPY staining was detected around the nucleus (DAPI) in differentiated spheroids in both lipedema and healthy spheroids. It is important to note the difference in nuclei distribution between undifferentiated and differentiated spheroids. Upon the induction of differentiation, the cells stop proliferating; whereas the undifferentiated cells located primarily on the periphery of the spheroid continue to divide resulting in rounded, compact sphere with a necrotic core. Adipogenesis was further confirmed by the expression of primary adipogenic marker genes (ADIPOQ, LPL, PPAR-γ, and Glut4). The data show a significant up-regulation in the expression of the ADIPOQ gene in healthy adipocyte-differentiated spheroids (*p* < 0.01) as compared to their corresponding undifferentiated spheroids (Figure 2B). Gene expression of the primary adipogenic genes PPAR-γ (*p* < 0.001), LPL (healthy *p* < 0.05; lipedema *p* < 0.001), and Glut 4 (healthy *p* < 0.01; lipedema *p* < 0.001), were significantly increased in differentiated spheroids in both groups compared to undifferentiated spheroids (Figure 2B,C). It is worth noting that no difference in adipogenic gene expression was detected between lipedema and healthy differentiated spheroids. 

### 2.3. Levels of Inflammatory Gene Expression in ASC Spheroids

**Figure 3:**
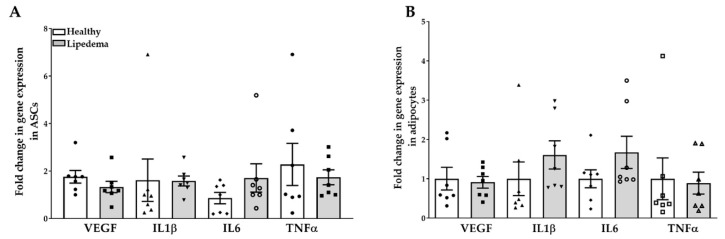
Expression of inflammation-associated genes in 3D spheroids derived from lipedema and healthy ASCs. The qRT-PCR data showed a similar gene expression of VEGF, IL-1β, IL-6 and TNFα in (**A**) undifferentiated and (**B**) differentiated 3D spheroids (*n* = 7 per group). The gene expression of the differentiated adipocyte spheroids is calculated from the percent of undifferentiated spheroids of each group. The results are displayed as scatter plots with bars. The values are the mean ± SEM. Abbreviations: VEGF: vascular endothelial growth factor; IL-1β: interleukin-1 beta; IL-6: interleukin-6; TNFα: tumor necrosis factor alpha.

ASCs isolated from lipedema patients and cultured in a 2D monolayer showed high levels of endogenous expression of inflammatory genes [33]. Thus, we determined the expression level of these genes in lipedema and healthy ASC spheroids by qRT-PCR. The data show an up-regulation of interleukin (IL)-6 gene expression in both lipedema ASCs (2-fold increase, *p* = 0.25) and adipocyte-differentiated spheroids (~1.8-fold increase, *p* = 0.23) compared to healthy spheroids (Figure 3A,B). However, no difference in the expression of the vascular endothelial growth factor (VEGF), tumor necrosis factor (TNFα) or IL-1β genes was detected in any of the samples (Figure 3A,B). 

### 2.4. Fibrosis in ASC Spheroids

**Figure 4:**
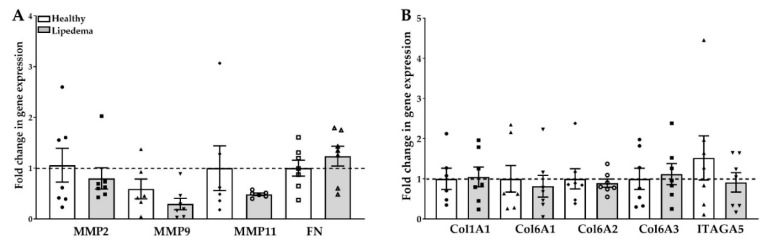
Expression of extracellular matrix (ECM) genes in lipedema- and non-lipedema-differentiated 3D spheroids. The qRT-PCR data showed a similar gene expression of (**A**) MMP-2, 9 and 11 and FN (*n* = 7 per group) and (**B**) Col1A1, Col6A1, Col6A2, Col6A3 and ITAGA5 (*n* = 7 per group). The gene expression of the differentiated adipocyte spheroids is calculated from the percent of undifferentiated spheroids of each group. The results are displayed as scatter plots with bars. The values are the mean ± SEM. The dotted line represents the expression level normalized to that of the undifferentiated spheroids. Abbreviations: Col: collagen; FN: fibronectin; ITAGA5: integrin A5; MMP: matrix metalloproteinase.

Collagen deposition, detected in lipedema AT, is tightly regulated by MMPs; therefore, determining the expression of the different collagen isoforms, fibronectin and integrin association in 3D spheroids is important in assessing fibrosis in vitro. The data show a decrease in gene expression of MMP2, MMP9 and MMP11 in lipedema-differentiated spheroids as compared to lipedema-undifferentiated and healthy spheroids (Figure 4A). However, no change in gene expression of either Cola1A1, Col6A1-3, FN, or integrin A5 was noted in any of the samples (Figure 4A,B).

### 2.5. Expression of ECM Components in 3D Differentiated Spheroids

**Figure 5:**
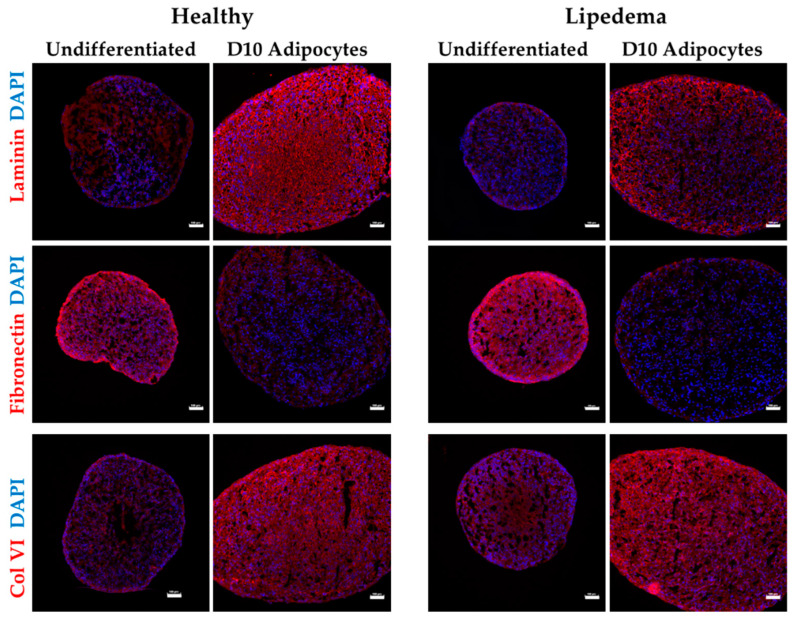
Characterization of ECM components of ASC spheroids. Immunofluorescence staining showing increase in the expression of laminin and Col VI and a decrease in the expression of fibronectin in Day10-differentiated spheroids compare to undifferentiated spheroids. Nuclei were stained with DAPI (blue; scale bar 100 μm).

Next, the protein expression of the primary ECM components in ASC spheroids was investigated using immunohistochemical staining. Laminin, collagen VI, and fibronectin have been detected in undifferentiated and adipogenic-differentiated spheroids of both lipedema and healthy cells. However, differentiated spheroids showed higher expression levels of laminin and Col VI, whereas fibronectin expression was significantly decreased as compared to the undifferentiated spheroids (Figure 5). It is worth noting that lipedema-differentiated spheroids expressed a lower level of laminin as compared to healthy cells; however, the quantitative analysis of the fluorescence intensity by NIS-Elements AR software revealed no statistically significant difference between the two groups (Figure S1).

### 2.6. Retention of Stemness Properties in ASC-Derived from 3D Spheroids

**Figure 6:**
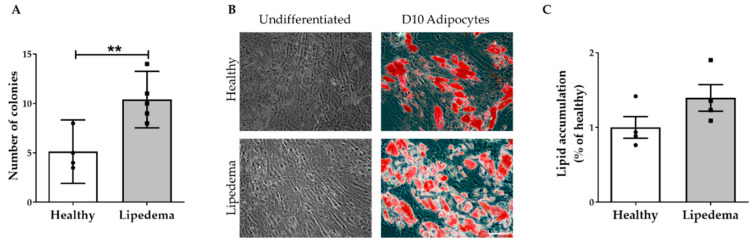
Characterization of ASC derived from 3D spheroids. (**A**) Quantitative analysis of the colony-forming unit fibroblast (CFU-F) assay revealed a significant increase in the CFU potential of ASCs from lipedema patients compared with healthy individuals (healthy *n* = 4; lipedema *n* = 5). The results are displayed as scatter plots with bars. The values are the mean ± SEM. ** *p* < 0.01. (**B**) Representative photomicrographs of the adipogenic differentiation of ASCs determined by Oil Red O staining 14 days after the induction adipogenesis. Lipid droplets (red) observed inside differentiated cells (scale bar, 100 μm). (**C**) The quantitative analysis of Oil Red O stain by spectrophotometric analysis showing a trend of an increase in the adipogenic differentiation potential of lipedema ASCs compared to healthy ASCs (*n* = 4 per group). The results are displayed as scatter plots with bars. The values are the mean ± SEM.

To further characterize ASC spheroids, cells were re-derived from trypsinized spheroids and re-plated in 2D monolayer cultures. The cultured-expanded ASCs retained their fibroblastic morphology and stemness characteristics, as observed in monolayer cultures. Their self-renewal ability assessed by colony-forming unit fibroblast (CFU-F) assay showed a significant increase in colonies in ASCs isolated from lipedema 3D spheroids as compared to healthy spheroids (Figure 6A). Moreover, ASCs derived from both lipedema and healthy 3D spheroids were able to differentiate to adipocytes once cultured in adipogenic-induced media (Figure 6B) with a trend of an increase in the adipogenic differentiation of lipedema ASCs compared to healthy ASCs (Figure 6C).

## 3. Discussion

ASC spheroids have demonstrated advantageous therapeutic potential over 2D-cultured ASCs due to their increased anti-inflammatory, anti-apoptotic and antitumor effects as well as enhanced stemness properties and multi-lineages differentiation capacities in vitro and in vivo co-culture models [15,45,46,47]. Studies have also shown that ASC spheroid’s chemotactic activity was improved due to the expression of adhesion molecules on their surfaces, which is crucial for tissue repair [14,17,47,48]. In this study, the data demonstrate that ASCs derived from lipedema and healthy individuals can self-assemble into spheroids within 24h of culture using a liquid overlay technique. This technique is widely used to study the stem cell niche in 3D mimicking the in vivo microenvironment that consists of cells, ECM, and secreted autocrine and paracrine factors [49,50,51]. In lipedema, these intra-cellular interactions involve endothelial, immune cells, the basement membrane and adhesion proteins such as integrins. The results demonstrate that ASC spheroids generated from both lipedema and healthy ASCs retain their stemness properties in 3D suspension cultures and differentiate into adipocytes and osteocytes (data not shown). Consistent with the data published by Hoefner et al., 2019 [52], ASC spheroids fully differentiated into adipocytes by day 10 of culture, as indicated by lipid accumulation in the cells, which is a shorter time period than the conventional 21 days required for 2D monolayer cultures. In contrast to the previous study, however, a significant decrease in the volume of non-induced ASC spheroids at day 10 in both groups was found, which suggests some sort of compaction occurs. The compaction of spheroids is attributed to a number of dynamic processes: (a) cytoskeleton reorganization forced by cellular self-assembly resulting in the formation of the spheroids; (b) cellular adhesion generated between cells and between cells and extracellular matrix which is imperative in molecular signaling pathways; and (c) cellular growth creating proliferating, quiescent and necrotic zones within the spheroids resembling an in vivo-like microenvironment effective in drug screening and development. These processes alone or in combination control the formation of spheroids and thus determine their characteristics.

Adipogenic differentiation of ASC spheroids was further confirmed by a significant increase in the expression of the main adipogenic genes, ADIPOQ, LPL, PPAR-γ, and Glut4 as compared to undifferentiated ASC spheroids in both groups. However, no difference in the adipogenic gene expression was detected between lipedema and healthy differentiated spheroids. In contrast to our previously published data in 2D monolayer cultures [36], no statistically meaningful difference in the adipogenic gene expression was detected between lipedema and healthy differentiated spheroids. This discrepancy may be the result of the culture conditions; 3D spheroids comprise proliferating, quiescent and possibly necrotic cells resulting in differential gene and protein expression as compared to 2D monolayer cultures. 

Furthermore, no changes in the expression of the inflammatory mediator genes were detected in ASCs undifferentiated and differentiated spheroids of both groups in a similar manner to the expanded ASCs in 2D monolayer culture (published data) [36].

The ECM plays a significant role in many aspects of stem cell regulation, differentiation, and development. In this study, we determined the expression levels of the primary ECM components in ASC spheroids [53,54,55]. The data demonstrate that both undifferentiated and adipogenic-differentiated spheroids express fibronectin, collagen 1A1 and collagen 6 isoforms (A1-3); although there was no difference in the expression of ECM components between lipedema and healthy spheroids, a decrease in gene expression for MMPs, in particular MMP11, in lipedema spheroids was noted. Although, the decrease in MMP11 is not statistically significant, the data are promising as it has been reported that MMP11 expression is associated with collagen deposition, degradation of collagen 6A3 and reduction in adipocyte function and metabolism in differentiated adipocytes [56,57,58]. Thus, a potential link between decreased MMP11 expression and the increase in fibrosis and adipogenesis observed in lipedema adipose tissue may have been defined. The expression of the collagen VI, fibronectin and laminin proteins as detected by immunohistochemistry indicate that adipogenic-differentiated spheroids express high levels of these essential basement membrane components surrounding adipocytes as compared to the undifferentiated lipedema and healthy spheroids. However, fibronectin expression was decreased in differentiated spheroids in both groups. Our data are consistent with published studies reporting changes in ECM makers in ASCs in both 2D and 3D cultures [59].

One of the primary concerns of ASC expansion is the retention of their stem cell properties and therapeutic properties in vitro. In this study, the data show that ASC spheroids maintained their stemness while in suspension (day 10) as confirmed by the expression of Nanog and Oct4 genes in a similar manner to monolayer cultures. In addition, ASCs derived from the 3D spheroids retained their fibroblastic morphology, stem cell characteristics and differentiation capabilities once re-plated in 2D monolayer culture. Interestingly, lipedema ASCs derived from 3D spheroids showed a significant increase in colony formation and in adipogenic differentiation potential as compared to the same cells derived from healthy spheroids, which is consistent with our previously published data on adipogenic differentiation of cultured ASCs in 2D monolayers [36]. Although ASC spheroids retain their stemness properties, one of the issues for future studies is the optimization of the 3D culture conditions such as the nutrients and oxygen diffuse throughout the entire spheroid to permit cell survival in vitro before transplantation. 

In conclusion, further characterization of lipedema ASCs in 3D cultures will lead to a deeper understanding of the stem cell microenvironment in lipedema tissue, which in turn may help explain how the tissue expansion (proliferation of progenitors/ASCs and differentiation into hypertrophic adipocytes) and fibrosis (collagen deposition) occurs and to possibly elucidate the interplay between adipogenesis, inflammation and angiogenesis in tissue development. Finally, lipedema ASC 3D spheroids will be used as a model to study cellular interaction with immune cells and endothelial cells creating a microenvironment that closely mimics the adipose tissue. This model may be useful for drug screening and pre-clinical trials, thus bridging the gap between 2D monolayer cultures and an in vivo model. 

## 4. Materials and Methods

### 4.1. Cell Culture

The ASCs utilized to generate spheroids were isolated from lipoaspirates of subcutaneous thigh adipose tissue from healthy and lipedema patients and characterized for their stemness in our previous work [33]. ASCs (passage 3) were cultured in Dulbecco’s Modified Eagle’s Medium/Ham’s F-12 (DMEM/F-12; Gibco, Gaithersburg, MD, USA) with 10% fetal bovine serum (FBS, Hyclone, Logan, UT, USA), and 1% antibiotic/antimycotic (Thermo Fisher Scientific, Waltham, MA, USA) maintained at 37 °C, 5% CO_2_. Table 1 summarizes the biologic characteristics of ASCs used in this study. 

### 4.2. Formation of ASC Spheroids 

ASCs were seeded at a density of 30 × 10^3^ cells/cm^2^ in 12-well plates coated with Ultrapure Agarose solution (1.5% *w*/*v*, Technologies, Carlsbad, CA, USA) dissolved in basal medium DMEM/F-12. The plates were placed on an orbital shaker at 50 rpm. ASCs aggregated in suspension and the assembly process was examined by microscopy (Nikon Eclipse TE200) at days 2 and 10 and images were captured using a Nikon Digital Camera DXM1200F and Nikon ACT-1 software version 2.7 at 10× magnification (Nikon, Melville, NY, USA). The diameter of the spheroids was measured by ImageJ software (National Institutes of Health, Bethesda, Maryland, USA, http://imagej.nih.gov/ij/).

### 4.3. Adipogenic Differentiation of ASC Spheroids 

After 2 days of ASCs in growth medium, adipogenic differentiation was induced using a commercial adipogenic differentiation medium (AdipoQual, Obatala Biosciences, New Orleans, LA, USA). ASC spheroids were cultured in adipogenic differentiation-inducing medium for differentiation of the cells or kept in DMEM/F12 medium for control undifferentiated cells. Media were changed every 3 days until day 10. 

### 4.4. RNA Isolation and Quantitative Real-Time PCR (qRT-PCR) 

Total RNA from undifferentiated ASCs and from ASCs that had undergone adipogenic differentiation was extracted using RNA extraction kit (Qiagen, Germantown, MD, USA) and then digested with DNase I (Qiagen)). A total of 1 μg of mRNA was used for cDNA synthesis with an Applied Bioscience purification kit (Thermo Fisher Scientific, USA). qRT-PCR was performed using the SYBR Green qPCR SuperMix (Bio-Rad, Hercules, CA, USA) according to the manufacturer’s instructions. Oligonucleotide primers were designed with the vendor’s software (IDT, USA). Table 2 lists the primer sequences used for qRT-PCR. PCR conditions were: 2 min at 95 °C, and 40 cycles of 15 s at 95 °C and 30 s at 60 °C. The target and reference genes were amplified in separate wells. All reactions were performed in duplicate. The 2^−ΔΔCT^ method was used to quantify gene expressions and data were normalized to GAPDH, which was used as an internal control.

### 4.5. Preparation of Frozen Sections and Immunofluorescence

ASC spheroids were fixed with 4% paraformaldehyde (PFA) for 30 min at room temperature (RT). Following fixation, spheroids were washed 3× with 1X phosphate-buffered saline (PBS), and embedded in optimal cutting temperature compound (OCT, Tissue–Tek; Thermo Fisher Scientific, Waltham, MA, USA) on dry ice and stored at −80 °C. For neutral lipid visualization, ASC spheroid cryosections (10 μm) were stained with boron-dipyrromethene (BODIPY; 2 μM, Invitrogen, Waltham, MA, USA) for 15 min at 37 °C. Slides were then mounted and coverslipped in VECTASHIELD antifade mounting medium with 4′,6-diamidino-2-phenylindole (DAPI; Vector Lab, Burlingame, CA, USA). For ECM staining, ASC spheroid cryosections were blocked with 1% bovine serum albumin (BSA, Sigma-Aldrich, St. Louis, MO, USA) for 30 min at RT, and then incubated with antibodies against collagen VI, laminin, and fibronectin. All antibodies were purchased from abcam (Cambridge, MA, USA). Following incubation, slides were washed 2× in 1XPBS and incubated with secondary antibody (Life Technologies, Carlsbad, CA, USA) for 1 h at RT. Slides were then mounted and coverslipped in VECTASHIELD Antifade Mounting Medium with DAPI and stored in the dark at 4 °C until imaging. Images were acquired using Eclipse Ti-300 microscope at 10× magnification (Nikon Instruments, Melville, NY, USA) and the analysis was performed using the NIS-Elements AR software (Nikon Instruments).

### 4.6. Characterizing ASCs Derived from 3D Spheroids

ASC spheroids cultured in suspension for four days were passaged and plated in 2D monolayer cultures. The cultured-expanded ASCs were seeded for colony-forming unit fibroblast (CFU-F) assay and differentiation experiments. For CFU-F assay, ASCs were seeded at a density of 0.5 × 10^2^ cells/cm^2^ per well in a 6-well plate and were cultured in 2D for 2 weeks. The medium was changed on day seven after cell seeding. At day 14, cells were washed 2× with 1× PBS and stained with 1ml of 3% crystal violet (Sigma, St. Louis, MO, USA) for 30 min at RT. The plates were then washed with deionized water and placed on the rocker for 10 min. The number of colonies was manually quantified, with only CFUs greater than 2 mm in diameter were recorded. Each experiment was performed in duplicate. For differentiation experiments, the cells were seeded at 2 × 10^4^ cells/cm^2^ and grown in DMEM/F-12 media to confluence. The cells were then cultured in adipogenic media for 14 days, followed by fixation and staining with Oil Red-O (Sigma, St. Louis, MO, USA). These cells were then visualized using Olympus CKX53 Inverted Microscope equipped with digital camera at 20× magnification (Olympus, Waltham, MA, USA). The absorbance of the eluted Oil Red O was measured at 584 nm wavelength by spectrophotometry. The differentiation values are reported as a percent of the undifferentiated control cells. 

### 4.7. Statistical Analysis

GraphPad PRISM 8 was used for all statistical analyses. Mann–Whitney test was used to determine the differences between the two groups of participants. One-way ANOVA followed by Tukey’s post hoc test was used to analyze the differences between the four groups. Asterisks (∗) indicate statistical significance: * *p* < 0.05; ** *p* < 0.01; *** *p* < 0.001.

## Figures and Tables

**Table 1:** Characteristics of healthy and lipedema patients. Lipedema stages are explained in detail in [30].

Characteristics	Healthy	Lipedema
N	8	8
Sex	Female	Female
Age	47.86 ± 2.28	44.57 ± 3.99
BMI	27.30 ± 1.042	30.43 ± 1.163
Stage 1	−	12%
Stage 2	−	63%
Stage 3	−	25%

**Table 2:** List of primers used for qRT-PCR.

Name	Forward (5′–3′)	Reverse (5′–3′)
IL-6	GTAGCCGCCCCACACAGACAGCC	GCCATCTTTGGAAGGTTC
LPL	GAGATTTCTCTGTATGGCACC	CTGCAAATGAGACACTTTCTC
IL-1 β	TCCCCAGCCCTTTTGTTGA	TTAGAACCA AATGTGGCCGTG
VEGF	AGGCCCACAGGGATTTTCTT	ATCAAACCTCACCAAGGCCA
Glut-4	AGC AGC TCT CTG GCA TCA AT	CAA TGG AGA CGT AGC ACA TG
Leptin	GAAGACCACATCCACACACG	AGCTCAGCCAGACCCATCTA
PPAR-γ	AGGCGAGGGCGATCTTG	CCCATCATTAAGGAATTCATGTCATA
GAPDH	CGCTGAGTACGTCGTGGAGTC	GCAGGAGGCATTGCAGATGA
TNF-α	GAG CCA GCT CCC TCT ATT TA	GGG AAC AGC CTA TTG TTC AG
Oct4	GACAGGGGGAGGGGAGGAGCTAGG	CTTCCCTCCAACCAGTTGCCCCAAAC
Nanog	AGTCCCAAAGGCAAACAACCCACTTC	TGCTGGAGGCTGAGGTATTTCTGTCTC
Col1 A1	CATGTTCAGCTTTGTGGACCTC,	AGGTGATTGGTGGGATGTCTT
Col6 A1	GACCTCGGACCTGTTGGGTAC	TACCCCATCTCCCCCTTCAC
Col6 A2	CTGCGACAAGCCACAGCAG	GGGCACACGATCTGAGGGT
Col6 A3	GAGCAGCTTGACAACATTGCC	GCCCAGAGCACTTGCAGG
ITAGA5	GACAGGGAAGAGCGGGCACTATGG	GTCCCTTCCCGGCCGGTAAAACTC
MMP-2	TTGACGGTAAGGACGGACTC	ACTTGCAGTACTCCCCATCG
MMP-9	TTGACAGCGACAAGAAGTGG	GCCATTCACGTCGTCCTTAT
MMP-11	ATTTGGTTCTTCCAAGGTGCTCAGT	CCTCGGAAGAAGTAGATCTTGTTCT
ADIPOQ	AACATGCCCATTCGCTTTAC	AGAGGCTGACCTTCACATCC
Fibronectin	TTCCTTGCTGGTATCATGGCA	TATTCGGTTCCCGGTTCCA

## Supplementary Materials

Supplementary materials can be found at https://www.mdpi.com/1422-0067/21/21/8350/s1. Figure S1: Characterization of ECM components of ASC spheroids. The quantitative analysis of the fluorescence intensity of ECM staining revealed no difference between healthy and lipedema in 3D differentiated spheroids (average of four fields/sample).

## Abbreviations

ASCs	Adipose tissue-derived stem cells
AT	Adipose tissue
ECM	Extracellular matrix
MMP	Matrix metalloproteinase
LN	Laminin
FN	Fibronectin
SVF	Stromal vascular fraction
CD	Cluster of differentiation
2D	Two-dimensional
3D	Three-dimensional
ADIPOQ	Adiponectin
LPL	Lipoprotein lipase
PPAR-γ	Peroxisome proliferator-activated receptor gamma
Glut4	Glucose transporter type 4
Lip	Lipedema
IL	Interleukin
VEGF	Vascular endothelial growth factor
TNFα	Tumor necrosis factor
Col	Collagen
CFU-F	Colony-forming unit fibroblast

## References

[1] Al-Ghadban S., Bunnell B.A. (2020). Adipose Tissue-Derived Stem Cells: Immunomodulatory Effects and Therapeutic Potential. Physiology.

[2] Gimble J.M., Katz A.J., Bunnell B.A. (2007). Adipose-Derived Stem Cells for Regenerative Medicine. Circ. Res..

[3] Gupta M., Ajay A.K. (2015). Fat on sale: Role of adipose-derived stem cells as anti-fibrosis agent in regenerative medicine. Stem Cell Res. Ther..

[4] Strong A.L., Neumeister M.W., Levi B. (2017). Stem Cells and Tissue Engineering: Regeneration of the Skin and Its Contents. Clin. Plast. Surg..

[5] Beeson W., Woods E., Agha R. (2011). Tissue Engineering, Regenerative Medicine, and Rejuvenation in 2010: The Role of Adipose-Derived Stem Cells. Facial Plast. Surg..

[6] Conese M., Annacontini L., Carbone A., Beccia E., Cecchino L.R., Parisi D., Di Gioia S., Lembo F., Angiolillo A., Mastrangelo F. (2020). The Role of Adipose-Derived Stem Cells, Dermal Regenerative Templates, and Platelet-Rich Plasma in Tissue Engineering-Based Treatments of Chronic Skin Wounds. Stem Cells Int..

[7] Alicka M., Major P., Wysocki M., Marycz K. (2019). Adipose-Derived Mesenchymal Stem Cells Isolated from Patients with Type 2 Diabetes Show Reduced “Stemness” through an Altered Secretome Profile, Impaired Anti-Oxidative Protection, and Mitochondrial Dynamics Deterioration. J. Clin. Med..

[8] Serena C., Keiran N., Ceperuelo-Mallafré V., Ejarque M., Fradera R., Roche K., Nuñez-Roa C., Vendrell J., Fernández-Veledo S. (2016). Obesity and Type 2 Diabetes Alters the Immune Properties of Human Adipose Derived Stem Cells. Stem Cells.

[9] Oñate B., Vilahur G., Camino-López S., Díez-Caballero A., Ballesta-López C., Ybarra J., Badimon L. (2013). Stem cells isolated from adipose tissue of obese patients show changes in their transcriptomic profile that indicate loss in stemcellness and increased commitment to an adipocyte-like phenotype. BMC Genom..

[10] Bunnell B.A., Flaat M., Gagliardi C., Patel B., Ripoll C. (2008). Adipose-derived stem cells: Isolation, expansion and differentiation☆. Methods.

[11] Gimble J.M., Bunnell B.A. (2011). Adipose-Derived Stem Cells Methods and Protocols.

[12] Mineda K., Feng J., Ishimine H., Takada H., Doi K., Kuno S., Kinoshita K., Kanayama K., Kato H., Mashiko T. (2015). Therapeutic Potential of Human Adipose-Derived Stem/Stromal Cell Microspheroids Prepared by Three-Dimensional Culture in Non-Cross-Linked Hyaluronic Acid Gel. Stem Cells Transl. Med..

[13] Mueller-Klieser W. (1997). Three-dimensional cell cultures: From molecular mechanisms to clinical applications. Am. J. Physiol. Physiol..

[14] Bartosh T.J., Ylöstalo J.H., Mohammadipoor A., Bazhanov N., Coble K., Claypool K., Lee R.H., Choi H., Prockop D.J. (2010). Aggregation of human mesenchymal stromal cells (MSCs) into 3D spheroids enhances their antiinflammatory properties. Proc. Natl. Acad. Sci. USA.

[15] Petrenko Y., Syková E., Šárka K. (2017). The therapeutic potential of three-dimensional multipotent mesenchymal stromal cell spheroids. Stem Cell Res. Ther..

[16] Park I.S., Rhie J.-W., Kim S.-H. (2014). A novel three-dimensional adipose-derived stem cell cluster for vascular regeneration in ischemic tissue. Cytotherapy.

[17] Cheng N.-C., Chen S.-Y., Li J.-R., Young T.-H. (2013). Short-Term Spheroid Formation Enhances the Regenerative Capacity of Adipose-Derived Stem Cells by Promoting Stemness, Angiogenesis, and Chemotaxis. Stem Cells Transl. Med..

[18] Frith J.E., Thomson B., Genever P.G. (2010). Dynamic Three-Dimensional Culture Methods Enhance Mesenchymal Stem Cell Properties and Increase Therapeutic Potential. Tissue Eng. Part C Methods.

[19] Chaicharoenaudomrung N., Kunhorm P., Noisa P. (2019). Three-dimensional cell culture systems as an in vitro platform for cancer and stem cell modeling. World J. Stem Cells.

[20] O’Donnell B.T., Al-Ghadban S., Ives C.J., L’Ecuyer M.P., Monjure T.A., Romero-Lopez M., Bunnell B.A. (2020). Adipose Tissue-Derived Stem Cells Retain Their Adipocyte Differentiation Potential in Three-Dimensional Hydrogels and Bioreactors (†). Biomolecules.

[21] Edmondson R., Broglie J.J., Adcock A.F., Yang L. (2014). Three-Dimensional Cell Culture Systems and Their Applications in Drug Discovery and Cell-Based Biosensors. ASSAY Drug Dev. Technol..

[22] Cai X., Xie J., Yao Y., Cun X., Lin S., Tian T., Zhu B., Lin Y. (2017). Angiogenesis in a 3D model containing adipose tissue stem cells and endothelial cells is mediated by canonical Wnt signaling. Bone Res..

[23] Morrison R.J., Nasser H.B., Kashlan K.N., Zopf D.A., Milner D.J., Flanangan C.L., Wheeler M.B., Green G.E., Hollister S. (2018). Co-culture of adipose-derived stem cells and chondrocytes on three-dimensionally printed bioscaffolds for craniofacial cartilage engineering. Laryngoscope.

[24] Rogan H., Ilagan F., Tong X., Chu C.R., Yang F. (2020). Microribbon-hydrogel composite scaffold accelerates cartilage regeneration in vivo with enhanced mechanical properties using mixed stem cells and chondrocytes. Biomaterials.

[25] Li M., Ma J., Gao Y., Dong M., Zheng Z., Li Y., Tan R., She Z., Yang L. (2020). Epithelial differentiation of human adipose-derived stem cells (hASCs) undergoing three-dimensional (3D) cultivation with collagen sponge scaffold (CSS) via an indirect co-culture strategy. Stem Cell Res. Ther..

[26] Resch A., Wolf S., Mann A., Weiss T., Stetco A.-L., Radtke C. (2018). Co-Culturing Human Adipose Derived Stem Cells and Schwann Cells on Spider Silk—A New Approach as Prerequisite for Enhanced Nerve Regeneration. Int. J. Mol. Sci..

[27] Tang H., Zhang Y., Jansen J.A., Beucken J.J.V.D. (2017). Effect of monocytes/macrophages on the osteogenic differentiation of adipose-derived mesenchymal stromal cells in 3D co-culture spheroids. Tissue Cell.

[28] Buso G., Depairon M., Tomson D., Raffoul W., Vettor R., Mazzolai L. (2019). Lipedema: A Call to Action!. Obesity.

[29] Al-Ghadban S., Herbst K.L., Bunnell B.A., Szablewski L. (2019). Lipedema: A Painful Adipose Tissue Disorder. Adipose Tissue—An Update.

[30] Herbst K.L., Feingold K.R., Anawalt B., Boyce A., Chrousos G., de Herder W.W., Dungan K., Grossman A., Hershman J.M., Hofland H.J., Kaltsas G. (2019). Subcutaneous Adipose Tissue Diseases: Dercum Disease, Lipedema, Familial Multiple Lipomatosis, and Madelung Disease.

[31] Suga H., Araki J., Aoi N., Kato H., Higashino T., Yoshimura K. (2009). Adipose tissue remodeling in lipedema: Adipocyte death and concurrent regeneration. J. Cutan. Pathol..

[32] Al-Ghadban S., Cromer W., Allen M., Ussery C., Badowski M., Harris D., Herbst K.L. (2019). Dilated Blood and Lymphatic Microvessels, Angiogenesis, Increased Macrophages, and Adipocyte Hypertrophy in Lipedema Thigh Skin and Fat Tissue. J. Obes..

[33] Felmerer G., Stylianaki A., Hägerling R., Wang A., Ströbel P., Hollmén M., Lindenblatt N., Gousopoulos E. (2020). Adipose Tissue Hypertrophy, An Aberrant Biochemical Profile and Distinct Gene Expression in Lipedema. J. Surg. Res..

[34] Felmerer G., Stylianaki A., Hollmén M., Ströbel P., Stepniewski A., Wang A., Frueh F.S., Kim B.-S., Giovanoli P., Lindenblatt N. (2020). Increased levels of VEGF-C and macrophage infiltration in lipedema patients without changes in lymphatic vascular morphology. Sci. Rep..

[35] Priglinger E., Wurzer C., Steffenhagen C., Maier J., Hofer V., Peterbauer A., Nuernberger S., Redl H., Wolbank S., Sandhofer M. (2017). The adipose tissue–derived stromal vascular fraction cells from lipedema patients: Are they different?. Cytotherapy.

[36] Al-Ghadban S., Diaz Z.T., Singer H.J., Mert K.B., Bunnell B.A. (2020). Increase in Leptin and PPAR-γ Gene Expression in Lipedema Adipocytes Differentiated in vitro from Adipose-Derived Stem Cells. Cells.

[37] Siems W., Grune T., Voss P., Brenke R. (2005). Anti-fibrosclerotic effects of shock wave therapy in lipedema and cellulite. BioFactors.

[38] Sun Y., Chen S., Zhang X., Pei M. (2019). Significance of Cellular Cross-Talk in Stromal Vascular Fraction of Adipose Tissue in Neovascularization. Arter. Thromb. Vasc. Biol..

[39] Kubo Y., Kaidzu S., Nakajima I., Takenouchi K., Nakamura F. (2000). Organization of extracellular matrix components during differentiation of adipocytes in long-term culture. Vitro Cell Dev. Biol. Anim..

[40] Mariman E.C.M., Wang P. (2010). Adipocyte extracellular matrix composition, dynamics and role in obesity. Cell. Mol. Life Sci..

[41] Nakajima I., Aso H., Yamaguchi T., Ozutsumi K. (1998). Adipose tissue extracellular matrix: Newly organized by adipocytes during differentiation. Differentiation.

[42] Datta R., Podolsky M.J., Atabai K. (2018). Fat fibrosis: Friend or foe?. JCI Insight.

[43] Chavey C., Mari B., Monthouel M.-N., Bonnafous S., Anglard P., Van Obberghen E., Tartare-Deckert S. (2003). Matrix Metalloproteinases Are Differentially Expressed in Adipose Tissue during Obesity and Modulate Adipocyte Differentiation. J. Biol. Chem..

[44] Lin D., Chun T.-H., Kang L. (2016). Adipose extracellular matrix remodelling in obesity and insulin resistance. Biochem. Pharmacol..

[45] Ceccarelli S., Pontecorvi P., Anastasiadou E., Napoli C., Marchese C. (2020). Immunomodulatory Effect of Adipose-Derived Stem Cells: The Cutting Edge of Clinical Application. Front. Cell Dev. Biol..

[46] Seo Y., Shin T.-H., Kim H.-S. (2019). Current Strategies to Enhance Adipose Stem Cell Function: An Update. Int. J. Mol. Sci..

[47] Zhang S., Liu P., Chen L., Wang Y., Wang Z., Zhang B. (2015). The effects of spheroid formation of adipose-derived stem cells in a microgravity bioreactor on stemness properties and therapeutic potential. Biomaterials.

[48] Ylöstalo J.H., Bartosh T.J., Coble K., Prockop D.J. (2012). Human Mesenchymal Stem/Stromal Cells Cultured as Spheroids are Self-activated to Produce Prostaglandin E2 that Directs Stimulated Macrophages into an Anti-inflammatory Phenotype. Stem Cells.

[49] Pennings S., Liu K.J., Qian H. (2018). The Stem Cell Niche: Interactions between Stem Cells and Their Environment. Stem Cells Int..

[50] Dorst N., Oberringer M., Grässer U., Pohlemann T., Metzger W. (2014). Analysis of cellular composition of co-culture spheroids. Ann. Anat. Anat. Anz..

[51] Metzger W., Sossong D., Bächle A., Pütz N., Wennemuth G., Pohlemann T., Oberringer M. (2011). The liquid overlay technique is the key to formation of co-culture spheroids consisting of primary osteoblasts, fibroblasts and endothelial cells. Cytotherapy.

[52] Hoefner C., Muhr C., Horder H., Wiesner M., Wittmann K., Lukaszyk D., Radeloff K., Winnefeld M., Becker M., Blunk T. (2020). Human Adipose-Derived Mesenchymal Stromal/Stem Cell Spheroids Possess High Adipogenic Capacity and Acquire an Adipose Tissue-like Extracellular Matrix Pattern. Tissue Eng. Part A.

[53] Nicolas J., Magli S., Rabbachin L., Sampaolesi S., Nicotra F., Russo L. (2020). 3D Extracellular Matrix Mimics: Fundamental Concepts and Role of Materials Chemistry to Influence Stem Cell Fate. Biomacromolecules.

[54] Gattazzo F., Urciuolo A., Bonaldo P. (2014). Extracellular matrix: A dynamic microenvironment for stem cell niche. Biochim. Biophys. Acta (BBA) Gen. Subj..

[55] Adapala V.J., Adedokun S.A., Considine R.V., Ajuwon K.M. (2012). Acute inflammation plays a limited role in the regulation of adipose tissue COL1A1 protein abundance. J. Nutr. Biochem..

[56] Gesta S., Guntur K., Majumdar I.D., Akella S., Vishnudas V.K., Sarangarajan R., Narain N.R. (2016). Reduced expression of collagen VI alpha 3 (COL6A3) confers resistance to inflammation-induced MCP1 expression in adipocytes. Obesity.

[57] Arcidiacono B., Chiefari E., Laria A.E., Messineo S., Bilotta F.L., Britti D., Foti D.P., Foryst-Ludwig A., Kintscher U., Brunetti A. (2017). Expression of matrix metalloproteinase-11 is increased under conditions of insulin resistance. World J. Diabetes.

[58] Dali-Youcef N., Hnia K., Blaise S., Messaddeq N., Blanc S., Postic C., Rio M.C. (2016). Matrix metalloproteinase 11 protects from diabesity and promotes metabolic switch. Sci. Rep..

[59] Kapur S.K., Wang X., Shang H., Yun S., Li X., Feng G., Khurgel M., Katz A.J. (2012). Human adipose stem cells maintain proliferative, synthetic and multipotential properties when suspension cultured as self-assembling spheroids. Biofabrication.

