# Supplementary material for: 3D Spheroids Derived from Human Lipedema ASCs Demonstrated Similar Adipogenic Differentiation Potential and ECM Remodeling to Non-Lipedema ASCs In Vitro
Source: Int J Mol Sci. 2020 Nov 7;21(21):8350. doi: 10.3390/ijms21218350 (PMC7664323; doi:10.3390/ijms21218350)
Supplement: Supplementary file 1 [file ijms-21-08350-s001.pdf]

3D spheroids derived from human lipedema ASCs demonstrated similar adipogenic differentiation potential and ECM remodeling to non-lipedema ASCs *in vitro*

Sara Al-Ghadban[1,2,], India A. Pursell[2], Zaidmara T. Diaz[2], Karen L. Herbst[3], Bruce A. Bunnell[1,2,*]*

[1]Department of Microbiology, Immunology and Genetics, University of North Texas Health Science Center, Fort Worth, TX, United States

[2]Center for Stem Cell Research and Regenerative Medicine, Tulane University School of Medicine, New Orleans, LA, United States

[3] Medical Director, Limitless Therapeutics, Los Angeles, CA, United States

*** Correspondence:** sara.al-ghadban@unthsc.edu; bruce.bunnell@unthsc.edu

Supplementary data

1. *Expression levels of ECM components in 3D differentiated spheroids*

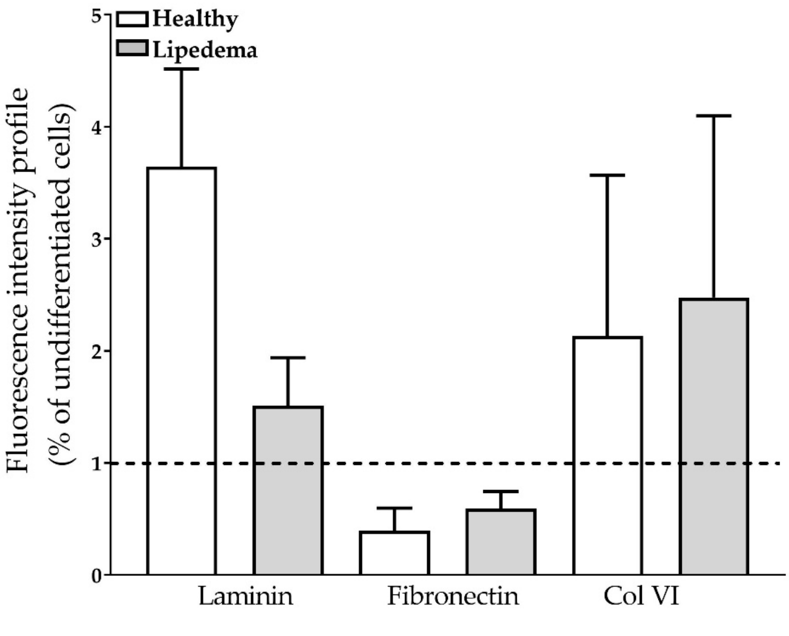

Figure S1: Characterization of ECM components of ASC spheroids. The quantitative analysis of the fluorescence intensity of ECM staining revealed no difference between healthy and lipedema in 3D differentiated spheroids (n=3 per group; average of 4 fields/sample). The dotted line represents the expression level normalized to that of the undifferentiated spheroids. Data are shown as mean ± SEM.
